# RUNX1 as a recombinase cofactor

**DOI:** 10.18632/oncotarget.5488

**Published:** 2015-07-11

**Authors:** Agata Cieslak, Dominique Payet-Bornet, Vahid Asnafi

**Affiliations:** Université Paris Descartes Sorbonne Cité, Institut Necker-Enfants Malades (INEM), Institut National de Recherche Médicale (INSERM) U1151, and Laboratory of Onco-Hematology, Assistance Publique-Hôpitaux de Paris (AP-HP), Hôpital Necker Enfants-Malades, Paris, France

**Keywords:** Immunology and Microbiology Section, Immune response, Immunity

The regulation of TCR rearrangements, particularly of TCRδ and TCRβ, plays a decisive role in lymphoid differentiation and oncogenic transformation. Somatic assembly of TCR loci is established through V(D)J recombination during lymphocyte development. All V, (D) and J gene segments are flanked with recombination signal sequences (RSS) which are composed of conserved heptamer and nonamer motifs separated by a non conserved spacer of either 12 or 23 base pairs. The V(D) J recombination process is initiated by the multimeric RAG1/RAG2 complex (RAG1/2), which binds to a 12RSS/23RSS pair (12/23 rule) and then introduces double-strand breaks (DSB) simultaneously at the two coding segment-RSS junctions. The coding joint is formed during the subsequent repair phase and this sequence is highly diverse and unique to each rearrangement.

Intrinsic RSS features had been reported to be directly involved in the control of V(D)J recombination beyond chromatin accessibility [1]. This has been clearly demonstrated for TCRβ gene assembly. Indeed, direct Vβ-Jβ rearrangement is prohibited by a mechanism operating beyond the 12/23 rule which imposes Dβ segment usage [2]. The B12/23 restriction imposes a two-step process for TCRβ assembly but does not explain the ordering (D-J before V-DJ). It has recently been proposed that Dβ 23RSS binds a transcription factor (TF), c-Fos, which efficiently recruits RAG1, ensuring that Dβ-Jβ rearrangement occurs first [3]. Unlike TCRβ, it was assumed that initial TCRδ locus rearrangements are not ordered but these conclusions were based essentially on murine data.

Given the importance of ordered recombination, extensive research has led to the identification of a critical role of RAG targeting or “RAG loading” to specific RSS by transcription factors [4]. In a recent report [5], we showed that human TCRδ gene rearrangements are strictly controlled by a Beyond 12/23 restriction involving the RUNX1 transcription factor, which behaves as a co-factor for the V(D)J recombinase: RUNX1 binds to *D*δ*2–23RSS*, interacts with RAG1 and enhances RAG1 deposition to *D*δ*2–23RSS* (Figure 1). Importantly, functional inactivation of RUNX1 led to the absence of normal Dδ2-Dδ3 rearrangements *in vivo,* while stable transfection of RUNX1 in a non-lymphoid cell line (BOSC23) lacking TCRδ rearrangement specifically induced Dδ2-Dδ3 recombination. Cieslak et al. also demonstrated that human TCRδ locus rearrangement is ordered, as the first rearrangement (Dδ2-Dδ3) occurs at a very immature thymic ETP (Early T-cell Precursor) CD34^+^/CD1a^−^ CD7^+dim^ stage and systematically precedes Dδ2(Dδ3)-Jδ1 rearrangement. We also showed that direct *D*δ*2* and *J*δ*1* joining is impeded by a B12/23 restriction and thus was never detected either *in vivo* or *in vitro*. Altogether the data of this study identified RUNX1 as a critical regulator of the earliest human TCRδ rearrangement by a protein-protein interaction involving RAG1. Interestingly, there is no RUNX1 binding site in the homologous murine Dδ RSS, suggesting that there may be fundamental differences between murine and human early T-cell ontogeny. Notably, this may provide a molecular explanation for the lack of ordered TCRδ gene assembly in mouse.

**Figure 1:**
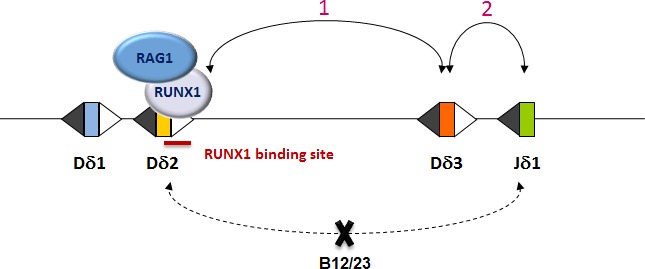
Dδ2-Jδ1 direct rearrangement is impeded by a B12/23 restriction, thus Dδ-Jδ assembly occurs in an ordered 2-steps process: Dδ2-Dδ3 rearrangement, which requires RUNX1, occurs first, then Dδ2Dδ3 rearranges with Jδ1.

RUNX1 imposes the use of two Dδ gene segments in all rearranged TCRδ chains during human TCRδ locus recombination. Why are two Dδ gene segments required for TCRδ chains? The D region encodes for the third complementary determining region (CDR3) which is a key element in antigen recognition. The usage of two Dδ gene segments is specific to TCRδ chain, and increases the diversity and the length of CDR3. After rearrangement, median CDR3 lengths are 14, 12 and 9 amino acids for TCRδ, IgH and TCRβ respectively [6]. This is an evolutionary advantage for human TCRγδ lymphocytes, which have potentially longer and more diversified TCRδ CDR3 than those seen in mice. Antigen recognition by TCRγδ is sometimes considered as ‘Antibody-like’ since unlike TCRαβ, it is not restricted to major histocompatibility complex (MHC). It is therefore possible that the CDR3d length may allow the γδTCR to escape the structural constraints imposed by MHC antigen presentation. Transcription factor-dependent recruitment of RAG to TCRδ RSSs offers unique opportunities for developmental control. With this in mind, it is worth noting that early human fetal thymus TCR delta rearrangements show little evidence of *D*δ*2* usage [7]. Based on this it is tempting to speculate that RUNX1 is responsible for a developmental shift from the early fetal to the postnatal pattern of TCRδ rearrangement.

The function of RUNX1 as a cofactor of V(D) J recombination provides novel insight into its role in normal lymphopoiesis and lymphoid oncogenesis. Indeed loss of function of RUNX1 in the very early stages of thymic maturation could lead to failure of initiation of TCRδ rearrangement, maturation arrest and the development of leukemia following the acquisition of additional mutational hits. This opens new perspectives in the comprehension of the role of *RUNX1* somatic mutations in AML FAB M0 and ETP T-ALL, which are characterized by blockage of the maturation in the early stages of development.

## References

[1] Krangel MS (2003). Nat Immunol.

[2] Bassing CH (2000). Nature.

[3] Wang X (2008). Nat Immunol.

[4] Jackson AM (2006). Immunol Rev.

[5] Cieslak A (2014). J Exp Med.

[6] Rock EP (1994). J Exp Med.

[7] Krangel MS (1990). J Exp Med.

